# Acupoint Activation: Response in Microcirculation and the Role of Mast Cells

**DOI:** 10.3390/medicines1010056

**Published:** 2014-11-20

**Authors:** Guangjun Wang, Daniela Litscher, Yuying Tian, Ingrid Gaischek, Shuyong Jia, Lu Wang, Weibo Zhang, Gerhard Litscher

**Affiliations:** 1 Institute of Acupuncture and Moxibustion, China Academy of Chinese Medical Sciences, 16 Nanxiaojie, Dongzhimennei, Beijing 100700, China; E-Mails: tjuwgj@gmail.com (G.W.); yytian803@aliyun.com (Y.T.); shuyong6666@163.com (S.J.); 2 Research Unit for Complementary and Integrative Laser Medicine, Research Unit of Biomedical Engineering in Anesthesia and Intensive Care Medicine, and TCM Research Center Graz, Medical University of Graz, Auenbruggerplatz 29, Graz 8036, Austria; E-Mails: daniela.litscher@medunigraz.at (D.L.); ingrid.gaischek@medunigraz.at (I.G.); lu.wang@medunigraz.at (L.W.)

**Keywords:** microcirculation, acupuncture point, mast cells

## Abstract

Background: According to Traditional Chinese Medicine (TCM) theory, acupuncture effects are based on the integrity function of meridians. Meridians are thought to regulate body function through the normal flow of qi and/or blood. Disturbances in this flow are thought to cause disease, and acupuncture techniques are believed to cure disease by regulating this flow. However, it is still difficult to understand the exact meaning of qi and to evaluate the activation of meridians. Thus, more and more attention has been focused on the relationship of acupuncture and circulation. Methods: In this narrative review, the authors focus on the state of the art in acupoint activation, microcirculation response, and on investigation of mast cells, based on current literature research. Results: Altogether, 52 references are cited and discussed critically. A schematic diagram of the relationship between acupuncture stimulation, changes of microcirculation and mast cells is presented as result. Conclusion: The block diagram presented in this review article shows that mast cells might play an important role in circulation response after acupoint stimulation.

## 1. Introduction

In Traditional Chinese Medicine (TCM) theory, acupuncture effects are based on the integrity function of meridians, so the meridian might be the core concept of metaphysical acupuncture theory [1]. Because the various meridians are thought to be connected, practitioners usually apply acupuncture along the pathway from one region to another. Meridians are thought to regulate body function through the normal flow of qi and/or blood. Disturbances in this flow are thought to cause disease, and acupuncture techniques are believed to cure disease by regulating this flow. Recently, the concept of deqi was explored from nervous function, and some people think qi is closely related to the nervous function [2,3,4,5,6,7], however, it is still difficult to understand the exact meaning of qi [8,9,10] and to evaluate the activation of meridians. Thus, more and more attention has been focused on the relationship of acupuncture and circulation [11,12,13]. In the meridian study area, the broad consensus in meridian study is a lower impedance along the meridians [14,15]. Usually, the impedance of the skin is proportional to the interstitial fluid volume which comes from microcirculation, so microcirculation might be an index for meridian activation [16]. On the other hand, some study results suggested that the meridian system might contain a continuous channel [17] to facilitate the signal transport in the peripheral tissues [16,18], providing further evidence for a relationship between microcirculation and meridians [19]. In this narrative review, we focus on the acupoint activation from the aspects of microcirculation.

## 2. Acupoint Stimulation and Corresponding Response in Microcirculation

In the research area of microcirculation, laser Doppler flowmetry (LDF) is widely being used for monitoring the microcirculation due to its advantage of a good frequency response, and is therefore well suited for noninvasive investigations of microvascular responses to acupuncture [11,20]. According to a previous study, the mean blood flow (MBF) was larger at the acupoints than in their surrounding tissues, which indicates that MBF can be used as an index for discriminating differences in the microcirculatory conditions between acupoints and their surrounding tissues [21]. It has also been shown that acupuncture cannot only increase general circulation [22] and circulation in specific organs [23], but it can also change the skin microcirculation as well [12,24,25,26]. When an acupoint was stimulated adequately, the blood perfusion of this point continued to increase, whereas the blood perfusion of a non-acupoint only changed slightly following the same stimulation [27]. These results indicated that the blood perfusion in acupoints can be recommended as a tool for the evaluation of acupuncture effects. One of the possible causes of acupuncture effects is the special sensation in an acupoint after stimulation, which might be related to blood perfusion changes in the acupoint or its meridian [28].

We know that many factors can influence the blood perfusion signal, such as heart rate, muscle contraction and others. Spectral analysis of LDF signals reveals that blood flow oscillations at frequencies from 0.009 to 1.6 Hz might reflect various physiological rhythms [29,30]. In a previous study, LDF signals were measured in healthy volunteers, and wavelet transformation with Morlet wavelet was applied. The results indicated that needling the Hegu (LI4) acupoint significantly increased the blood flow, significantly decreased the relative energy contribution at 0.02–0.06 Hz and significantly increased the relative energy contribution at 0.4–1.6 Hz at Hegu, but induced no significant changes at the non-acupoints. This is the first time that spectral analysis was used to investigate the microcirculatory blood flow responses induced by acupuncture stimulation, and revealed possible differences in sympathetic nerve activities between needling the Hegu acupoint and its nearby non-acupoint [31].

In our study, the results suggested that after stimulation of the right LI4 with laser needle, MBF in the left Hegu acupoint increased significantly, which is in accordance with our previous study [32,33]. On the other hand, non-acupoint stimulation had no effect in left LI4, which suggested that the laser needle effect might have specificity. A further analysis by Morlet wavelet analysis, as done previously [12,24,34], indicated that stimulation of the right LI4 acupoint only affected frequency bands 0.0095–0.02 Hz, 0.02–0.06 Hz and 0.06–0.15 Hz, which are influenced by endothelial activity, neurogenic activity and the intrinsic myogenic activity of vascular smooth muscles of the vessel wall, respectively [35]. These results indicate that there are many factors which can affect the blood flow, and the changes of blood flow induced by acupuncture stimulation can be specific.

In clinical practice, acupuncture effectiveness not only depends on the correct acupoint selection, but also on the correct manipulation. Different manipulations, such as reinforcing and reducing, might result in different effects. Recently, a study indicated that different manipulations resulted in different changes of blood perfusion at the Zusanli acupoint in healthy subjects [36]. Generally, skin temperature is related to blood perfusion. If the different acupuncture manipulations induce different changes of blood perfusion, they can also produce different temperatures in the body. Actually, previous results indicated that different manipulations result in different changes in skin temperature [37,38]. However, other mechanisms, like heat production in muscles and brown adipose tissue will counteract heat loss or heat gain via the skin.

## 3. Mast Cells Play an Important Role in Acupoint Activation and Microcirculatory Response

Previous results indicated that acupuncture can regulate microcirculation. In other words, circulation response may be regarded as an index of acupoint activation. However, acupuncture and the circulation response can be related via an intermediate bridge. Numerous studies indicated that mast cells might be the best candidate of this intermediate bridge.

Mast cells are generally considered to play a key role in the acute allergic reaction [39], the body's antimicrobial reaction [40,41], and parasite infection [42]. According to further research, the role of mast cells exceeds our traditional understanding [43,44]. Particularly, mast cells play an important role in acupuncture therapy [45,46,47,48].

**Figure 1:**
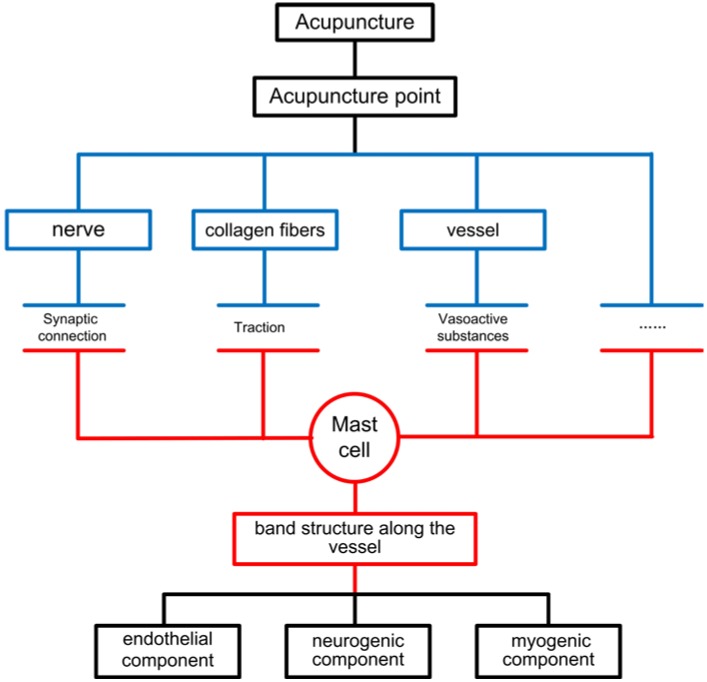
Block diagram of the relationship between acupuncture stimulation, changes of microcirculation, and mast cells.

Professor Yao’s work emphasized the role of mast cells in the function of meridians [19], which partly supported our work about low hydraulic resistance channels [16,49]. According to the low hydraulic resistance channel hypothesis, mast cells release vasoactive substances such as histamines, which regulate the vessel permeability. However, these vasoactive substances can also stimulate the mast cells to release more vasoactive substances. Similar to a positive feedback mechanism, the acupuncture signal can be transported along the meridian. These works only hypothesized with regard to function, and the work which finally fully described the relationship between mast cells and circulation is Luo’s morphological work [50]. In this work, a new dyeing method was described, which can observe blood vessels, Acetylcholinesterase (AchE) positive nerve fibers and mast cells at the same time. In the acupoint area, mast cells form a band structure along the vessels which is surrounded by the AchE-positive fibers. In particular, mast cells are concentrated at the vascular bifurcation, which means that mast cells can regulate the vessel with a high efficiency. On the other hand, numerous studies have suggested that mast cells form the synaptic connections with the nerve [51,52]. Thus, during acupuncture, active nerve fibers, traction from the collagen fibers and other factors can activate the mast cells, and through the function of the mast cell band along the vessel, the acupoint will be activated and the microcirculatory response can be observed (Figure 1).

## 4. Conclusions

Blood vessels, mast cells and AchE can be clearly observed in acupoint tissues [50]. The further investigation of dynamic changes of the microcirculatory system, the immune system and the nervous system will be important in future studies on acupuncture mechanisms.
